# Hemolysis associated with pneumatic tube system transport for blood samples

**DOI:** 10.12669/pjms.301.4228

**Published:** 2014

**Authors:** Hasan Kara, Aysegul Bayir, Ahmet Ak, Selim Degirmenci, Murat Akinci, Ahmet Agacayak, Emine Marcil, Melih Azap

**Affiliations:** 1 Hasan Kara, Selcuk University, Faculty of Medicine, Department of Emergency Medicine, Konya, Turkey.; 2 Aysegul Bayir, Selcuk University, Faculty of Medicine, Department of Emergency Medicine, Konya, Turkey.; 3 Ahmet Ak, Selcuk University, Faculty of Medicine, Department of Emergency Medicine, Konya, Turkey.; 4 Selim Degirmenci, Selcuk University, Faculty of Medicine, Department of Emergency Medicine, Konya, Turkey.; 5 Murat Akinci, Selcuk University, Faculty of Medicine, Department of Emergency Medicine, Konya, Turkey.; 6 Ahmet Agacayak, National Poison Information Center, Ankara, Turkey.; 7 Emine Marcil, Konya Numune Hospital, Department of Emergency Medicine, Konya, Turkey.; 8 Melih Azap, Konya Numune Hospital, Department of Emergency Medicine, Konya, Turkey.

**Keywords:** Analysis, Blood, Biochemistry, Hematology

## Abstract

***Objective:*** The frequency of hemolysis of blood samples may be increased by transport in a pneumatic tube system. The purpose of this study was to evaluate the effect of pneumatic tube system transport on hemolysis of blood samples.

***Methods:*** Blood samples were transported from the emergency department to the hospital laboratory manually by hospital staff (49 patients) or with a pneumatic tube system (53 patients). The hemolysis index and serum chemistry studies were performed on the blood samples and compared between the different methods of transport.

***Results:*** The blood samples that were transported by the pneumatic tube system had a greater frequency of hemolysis and greater mean serum potassium and median creatinine, aspartate aminotransferase, and lactate dehydrogenase levels than samples transported manually.

***Conclusion:*** Blood samples transported from the emergency department to the hospital laboratory by a pneumatic tube system may have a greater frequency of hemolysis than samples transported manually. This may necessitate repeat phlebotomy and cause a delay in completing the laboratory analysis.

## INTRODUCTION

Hospital care requires transport of numerous drugs, clinical specimens, medical instruments, radiographs, and laboratory reports between departments. This is costly to hospitals and requires the employment of hospital transportation staff. Pneumatic tube systems are available to transport clinical specimens, drugs, radiographs, and laboratory reports more rapidly and safely than manual transport by hospital workers.^1^ However, the transport method may affect the quality of samples.^2^ Rapid transport of blood samples in pneumatic tube systems may cause red blood cell breakdown (hemolysis) and may affect blood test results.^3^^,^^4^ Hemolysis of blood samples may necessitate repeated phlebotomy and may cause inaccuracy and delays in completing laboratory tests that are needed for clinical decision-making.^5^

Recently, the pneumatic tube system in our hospital was out of order. Blood samples from patients in the emergency department were transported manually by hospital staff from the emergency department to the laboratory. During this time, we observed that laboratory results frequently were normal, including the hemolysis index. When the pneumatic tube system was repaired and functional for transport of blood samples, the hemolysis index was higher. Therefore, we performed this study to evaluate the effect of the pneumatic tube system on hemolysis of blood samples. We hypothesized that the pneumatic tube system may cause hemolysis of blood samples that may necessitate repeat phlebotomy and cause delays in patient care.

## METHODS

***Study Design:*** This study was performed after the approval of the Ethic Board of Konya Numune Hospital. From 09.12.2011 to 15.12.2011, the hospital pneumatic tube system was not functioning, and blood samples were transported manually by hospital staff from the emergency department to the laboratory for evaluation of biochemical parameters; manually-transported blood samples were randomly selected from 49 patients for the study. From 16.12.2011 to 22.12.2011, the hospital pneumatic tube system was functional and used for transport of blood samples from the emergency department to the laboratory; blood samples that were transported by the pneumatic tube system were randomly selected from 53 patients for the study. In this study, all blood samples were taken from a single vein with an injection by a phlebotomist. The blood samples were inserted into the tubes containig no anticoagulant, heparin, K2EDTA or citrate (BD Vacutainer, SST II Advance, UK). While the samples taken in the first week were transferred from the emergency department to the central laboratory with the help of a porter, the samples obtained in the second week were trasferred through the pneumatic tube system. The blood samples of both groups were centrifuged at 3500 rpm for 10 min. Hemolysis index of the samples was determined with spectrophotometry. Other quantitative clinical biochemical studies included serum sodium, potassium, calcium, glucose, urea nitrogen, creatinine, alanine aminotransferase, aspartate aminotransferase, lactate dehydrogenase, total bilirubin, and amylase.

***Pneumatic tube system: ***Pneumatic tube system (Swisslog Compact Station, Germany) used in the hospital is running at 3m/sec. Our pneumatic tube system contains carriers with sponge to prevent the tubes to hit each other and enables them to move. These carriers are made ​​of polycarbonate in 86 mm diameter and 220 mm long, The distance of the pneumatic tube system between emergency department and central laboratory was approximately 80 meters. Since the emergency department and the central laboratory are on the ground floor of the hospital, the shortest distance of the pneumatic tube system was between emergency department and the central laboratory.

***Statistical analysis:*** Data analysis was performed with statistical software (Genstat 7, Release 7, VSN International, Hemel Hempstead, UK).^6^ Distribution properties of laboratory tests were evaluated with Anderson-Darling normality test. Normally distributed data were compared with t test. Data that were not normally distributed were compared with Mann-Whitney test. Hemolysis index of specimens transported with the pneumatic tube system and manual transport were compared with odds ratios. Statistical significance was defined by P ≤ 0.05. 

## RESULTS

The blood samples that were transported by the pneumatic tube system had a greater frequency of hemolysis and greater mean serum potassium and median creatinine, aspartate aminotransferase, and lactate dehydrogenase than samples transported manually (Table-I). The samples that were transported by the pneumatic tube system had hemolysis 532-fold more hemolysis (95% confidence interval: 30 to 9486) than samples that were transported manually. There were no other differences in serum blood parameters between samples transported by the pneumatic tube system or manually (Table-I).

**Table-I:** Relation between method of transport of blood specimens and results of laboratory studies *

*Parameters *	*Manual * *(n = 49)*	*Pneumatic tube system* *(n = 53)*	*P < *
Hemolysis (number [%])	8 (16)	53 (100)	0.0001
Sodium (mmol/L)	141 (140 to 142)	141 (139 to 142)	NS
Potassium (mmol/L)	4.3 ± 0.4	4.6 ± 0.5	0.001
Calcium (mg/dL)	9.4 ± 0.6	9.3 ± 0.6	NS
Glucose (mg/dL)	106 (93 to 123)	112 (95 to 161)	NS
Urea nitrogen (mg/dL)	32 (25 to 39)	35.50 (29.75 to 43.0)	NS
Creatinine (mg/dL)	0.80 (0.70 to 0.90)	0.90 (0.80 to 1.0)	0.001
Alanine aminotranferase (U/L)	18 (14 to 27)	18 (14 to 31)	NS
Aspartate aminotranferase (U/L)	19 (16 to 24)	27 (21 to 36)	0.0001
Lactate dehydrogenase (U/L)	190 (166 to 220)	284.5 (228 to 384)	0.0001
Bilirubin, total (mg/dL)	0.60 (0.45 to 0.80)	0.55 (0.40 to 1.03)	NS
Amylase (U/L)	59 (46 to 72)	66 (45 to 86)	NS

* Data reported as median (first and third quartile), mean ± SD, or number (%)

## DISCUSSION

The present results showed that samples transported by the pneumatic tube system had a markedly greater frequency of hemolysis than samples transported manually (Table-I). The pneumatic tube system may have caused hemolysis because of the high transportation speed, sudden changes in direction of the transport containers, and pressure caused by the system on the samples. Therefore, it may be prudent to monitor for hemolysis in blood samples transported by pneumatic tube systems. At installation of the pneumatic tube system, sudden changes in direction may be avoided. In addition, the speed and pressure of the system may be measured at regular intervals and optimized to minimize hemolysis of blood samples.

It is important that blood tests have sufficient accuracy for correct interpretation of results for clinical decision-making. Therefore, hospitals must address factors that may affect the accuracy of laboratory results. Hemolysis may be prevented by using gel in test tubes for blood collection.^7^ Furthermore, conveyor containers can be lined with cotton and protective circular rings to prevent hemolysis by minimizing acceleration and deceleration of samples during transport. 

Hemolysis may be caused by in vivo factors such as hemolytic anemia or blood transfusions or in vitro factors such as osmotic, physical, mechanical, or chemical factors. In the present study, the pneumatic tube system was associated with hemolysis of transported blood samples (Table-I). Abnormalities of potassium, magnesium, phosphate, aspartate aminotranferase, and lactate dehydrogenase have been observed previously in blood samples transported by pneumatic tube systems.^7^^-^^11^ The length of the pneumatic tube system and transfer speed may increase the pressure on erythrocytes and cause hemolysis.^12^ Hemolysis also may be caused by high speed, rapid acceleration, or rapid deceleration during sample transport in a pneumatic tube system.

In a previous study, the frequency of hemolysis differed between manual transport (16.2%) and a pneumatic tube system (13.7%).^3^ In another study, the frequency of hemolysis was greater with a pneumatic tube system (10.9%) than manual transport (3.3%), and failure of the pneumatic tube system caused in increase in the frequency of hemolysis (55%).^4^ The frequency of hemolysis observed in the present study (Table-I) was marked higher than previously reported. This difference between studies may have been caused by different characteristics of the pneumatic tube systems. Previous studies also showed changes in potassium, lactate dehydrogenase, iron, magnesium, and uric acid levels because of hemolysis from sample transport in a pneumatic tube system.^13^ The hemolysis index may be helpful to control the unity of samples and is a simple and inexpensive marker to quantify the quality of samples before analysis.^14^

Evliyaoglu et al. reported that the hemolysis rate of plasma samples increased very slowly as the pneumatic tube system transport rate and distance increased.^15^ In our study, although the distance between the emergency department and the central laboratory was the shortest, the hemolysis index was very high.

## CONCLUSIONS

The present study showed that blood samples transported by a pneumatic tube system may develop hemolysis during transport. Prudent quality control measures may include monitoring the hemolysis index of samples transported by the pneumatic tube system and evaluating the physical parameters of the system that may contribute to hemolysis.

## Notes

***Funding acknowledgement: ***This research received no specific grant from any funding agency in the public, commercial, or not-for-profit sectors.

## Notes

***Declaration of conflicting interests:*** The authors declare that there is no conflict of interest.
